# Investigation of the Intensity of Thrombocytosis as a Potential Prognostic Indicator in Canine Malignant Neoplasms

**DOI:** 10.1155/vmi/1271185

**Published:** 2026-05-21

**Authors:** Letícia Gondim Souto, Fwu Shing Teng, Susana Eduardo Vieira, Regina Kiomi Takahira

**Affiliations:** ^1^ Veterinary Clinic Department, São Paulo State University (UNESP)-School of Veterinary Medicine and Animal Science, Botucatu, Brazil

**Keywords:** cancer, metastasis, platelets, survival

## Abstract

Platelets contribute to cancer progression, and thrombocytosis is a recognized poor prognostic factor in human oncology; however, its significance in veterinary medicine remains underexplored. We retrospectively analyzed 166 dogs diagnosed with neoplasia and thrombocytosis (platelet count ≥ 430,000/μL) to assess the relationship between thrombocytosis intensity and survival. Survival analyses revealed that compared with dogs with mild thrombocytosis, those with moderate‐to‐severe thrombocytosis had significantly shorter survival times. With respect to specific diagnoses, dogs with mammary carcinoma and moderate‐to‐severe thrombocytosis had a significantly higher risk of death (HR = 3.4; *p* = 0.02). A similar trend was observed in patients with mast cell tumors, where the risk of death was 7.39 times higher, although this finding reached only borderline statistical significance (*p* = 0.05). Further prospective studies with larger cohorts are warranted to validate thrombocytosis as a prognostic biomarker and to elucidate its role in tumor biology and clinical outcomes in veterinary oncology.

## 1. Introduction

Platelets are often viewed as cytoplasmic fragments involved only in hemostasis and thrombosis. However, in human medicine, another critical role has become evident: their contribution to cancer progression.

For this reason, platelets have emerged as a significant area of interest for prognostic and therapeutic investigations [1, 2] since complex mechanisms and molecular interactions between neoplastic cells and circulating platelets have been revealed, highlighting the influential role of platelets in neoplastic development and metastasis [3].

One of the main mediators of reactive thrombocytosis is interleukin‐6 (IL‐6) [4]. This cytokine is secreted by tumor cells, which stimulate the liver to produce thrombopoietin (TPO) and, consequently, to stimulate megakaryopoiesis and platelet genesis, promoting an increase in the circulating platelet count [5]. The combination of these events contributes to thrombocytosis and hypercoagulability in cancer patients, known as Trousseau syndrome [6].

Platelet activation promotes the release of alpha granules, resulting in the stimulation of various factors that play crucial roles in the tumor microenvironment. These elements can influence fundamental aspects of tumors, such as cell growth, metastasis, angiogenesis, inflammation, and even the effectiveness of chemotherapy [7].

These factors include vascular endothelial growth factor (VEGF), transforming growth factor (TGF‐β), epidermal growth factor (EGF), and platelet‐derived growth factor (PDGF), which promote the growth, proliferation, and differentiation of tumor cells [8]. High serum levels of PDGF are associated with a significantly greater degree of metastasis and shorter survival in patients with breast cancer [9] and have been proposed as prognostic markers in patients with colorectal cancer [10].

Concomitantly, physical interactions between cancer cells and platelets can occur, which are mediated primarily by the adhesion molecule P‐selectin. This interaction not only provides a physical barrier that protects cancer cells but also interferes with their recognition by natural killer (NK) cells [11]. Additionally, platelets can transfer class I major histocompatibility complex (MHC) molecules to the surface of cancer cells, rendering them unrecognizable to the immune system and thereby impairing NK cell cytotoxicity and reducing IFN‐γ production [12].

Thrombocytosis in human cancer patients is widely recognized as a poor prognostic factor and is characterized by an inverse correlation between increased platelet count and patient survival [13]. Studies have shown that this condition is associated with various types of cancer, including hepatic neoplasms [14], gastrointestinal cancers [15], and cancers of the female reproductive system, such as cervical [16] and ovarian neoplasms [17].

Current research is focused on understanding whether reducing platelet counts and/or inhibiting their function could block platelet‐associated tumor progression [18, 19].

Tao et al. [20] reported that medications that reduce platelet count or platelet activation may decrease cancer progression in humans and improve patient survival outcomes. In a study by Borsig et al. [21], human patients who used aspirin for cardiovascular diseases presented a reduced risk of metastasis. Additionally, experimental models have demonstrated that antiplatelet therapy with heparin reduces the likelihood of developing metastatic disease [22].

This association in veterinary medicine is rarely documented or potentially underreported [23]. On the basis of retrospective studies in animals, thrombocytosis has been shown to be a common condition in dogs diagnosed with neoplasia [24–28], although its prognostic value across different types of neoplasms remains undetermined.

This research was motivated by the lack of information regarding the survival of dogs with neoplasia in relation to the severity of thrombocytosis across different neoplastic origins and behaviors. This gap in knowledge highlights the potential for future studies to guide new therapeutic interventions aimed at improving the survival of affected animals.

This study aimed to determine whether there is a relationship between the severity of thrombocytosis in canine patients with neoplasia and survival time, similar to what has been observed in human patients.

## 2. Methods

All procedures were approved by the Ethics Committee on Animal Use (CEUA) of the School of Veterinary Medicine and Animal Science at São Paulo State University (CEUA/UNESP #0202/2024).

### 2.1. Data Collection

Medical records of canine patients from the Veterinary Hospital (HV) of the School of Veterinary Medicine and Animal Science–UNESP/Botucatu were requested for the period between 2014 and 2023. The records selected included cases with thrombocytosis (> 430,000/μL) [29] and animals with cytopathological and/or histopathological diagnoses of neoplasia. The first hemogram performed at the HV of the patient with suspicion of neoplasia was considered for the statistical data.

The exclusion criteria included the following:

• Pharmacological or surgical treatments affecting platelet count:
  ◦ Use of corticosteroids or chemotherapy.
  ◦ Splenectomy.
• Incomplete or missing survival data.
• Animals that died from causes unrelated to the neoplasia.
• Neoplasms not fitting the proposed cytotypes (epithelial, mesenchymal, and round cell tumors).
• Animals are diagnosed with endocrine disorders, metabolic diseases, or other conditions potentially causing clinically relevant hemostatic alterations.

Survival time was defined as the interval from the date of neoplasia diagnosis to the date of death or last known follow‐up.

### 2.2. Platelet Count

Platelet counts were determined by automated hematology analysis and confirmed by blood smear evaluation performed by a veterinary clinical pathologist.

### 2.3. Data Organization and Statistical Analysis

Malignant cases were analyzed using survival time in months. Platelet counts were based on a modified version of the criteria by Woolcock et al. [27]. Due to sample size constraints that limited a four‐tier analysis, the original classification was collapsed into two categories: mild (> 430,000 and ≤ 600,000/μL) or moderate–severe (> 600,000/μL).

Overall survival was estimated using the Kaplan‒Meier method and compared with the log‐rank test. Multivariate Cox proportional hazards models were used to evaluate the prognostic impact of thrombocytosis. Proportional hazards assumptions were evaluated using the Schoenfeld test.

Hazard ratios (HRs) and their corresponding confidence intervals were calculated. Statistical significance was defined as a *p* value < 0.05.

All analyses were conducted using R software (Version 4.4.1). The raw data and all Cox proportional hazards models are provided in the supporting data (Tables S1 and S2), and the analysis codes are available at https://github.com/TengFwu/Statistical_analysis_Gondim_Souto_2025.

## 3. Results

### 3.1. Prevalence

In this study, the diagnosis of neoplasms (*n* = 166) was primarily through histopathological examination (107 cases), followed by cytopathological examination (59 cases).

On the basis of biological behavior, approximately 141 cases were classified as malignant, while 22 cases were classified as benign. Additionally, three patients lacked a conclusive determination of their biological behavior because of diagnostic limitations, such as cytopathology or the need for complementary tests, such as immunohistochemistry.

From the perspective of cytotype and/or histogenesis, 89 cases were of epithelial origin, 45 cases were of mesenchymal origin, and 23 cases were of round cell origin.

Epithelial neoplasms represented 46.3% (77 cases) of the cases with malignant behavior, 6.02% (10 cases) with benign behavior, and 1.2% (2 cases) without defined biological behavior. Among the mesenchymal neoplasms, 21.6% (36) were malignant, and 5.4% (9 cases) were benign. Round cell neoplasms comprised 11.4% (19) of the cases with malignant behavior and three benign cases, all of which were cytopathologically diagnosed as transmissible venereal tumors (TVTs).

Most cases (85%) were screened for metastasis, primarily through diagnostic imaging (e.g., radiography and computed tomography). Evidence of metastasis was detected in 39 cases, predominantly in epithelial neoplasms (21/39).

Epithelial neoplasms had the highest prevalence, representing 53.6% (89 cases), with mammary neoplasms accounting for approximately 50% of these cases, followed by squamous cell carcinomas (SCCs) (15.7%). Mesenchymal neoplasms represented 27.1% (45 cases), with vascular neoplasms, such as hemangiomas and hemangiosarcomas, accounting for 26.6%, and sarcomas accounting for 17.7%. Round cell neoplasms accounted for 13.8% (23) of the cases, predominantly mast cell tumors (69.5%).

Among the most prevalent neoplasms in this study, mammary carcinoma had the highest occurrence (45 cases), followed by mast cell tumors (16 cases), SCC (14 cases), sarcoma (11 cases), and hemangiosarcoma (10 cases).

### 3.2. Assessment of the Effect of Thrombocytosis on Survival Time in Patients With Malignant Neoplasms

When all malignant cases were grouped by the intensity of thrombocytosis, Kaplan–Meier analysis revealed that animals with mild thrombocytosis had significantly longer survival (median 44.1 months; 95% CI: 35.4–not estimable) than those with moderate‐to‐severe thrombocytosis did (median 15.1 months; 95% CI: 4.5–56.3; log‐rank *p* = 0.0014) (Figure 1(a)).

FIGURE 1Kaplan–Meier survival curves for dogs with all malignant tumors (a) and malignant tumors diagnosed by histopathology only (b), stratified by the intensity of thrombocytosis. Dogs with mild thrombocytosis (green line) demonstrated significantly longer overall survival than dogs with moderate‐to‐severe thrombocytosis did (orange line). The difference in survival distributions was statistically significant (log‐rank test; A: *p* = 0.0014; B: *p* = 0.0097).

**Figure:**
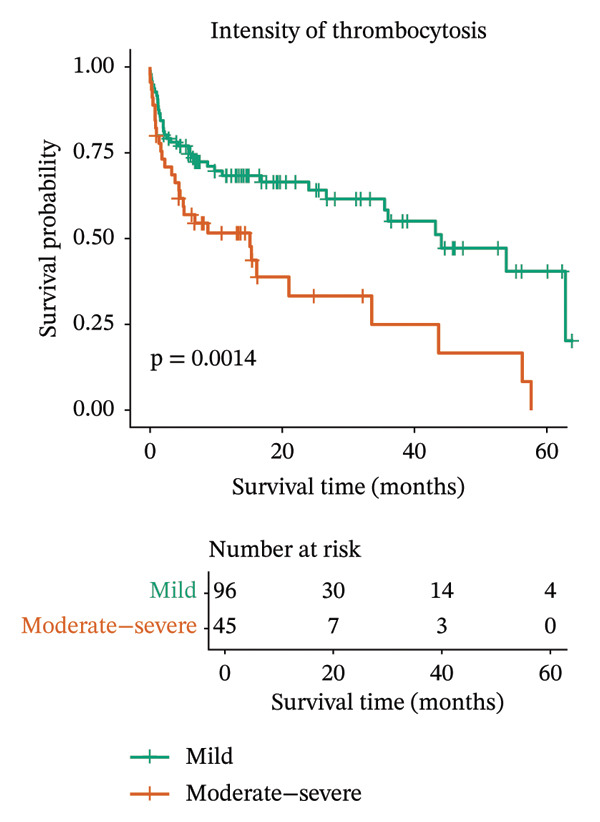
(a)

**Figure:**
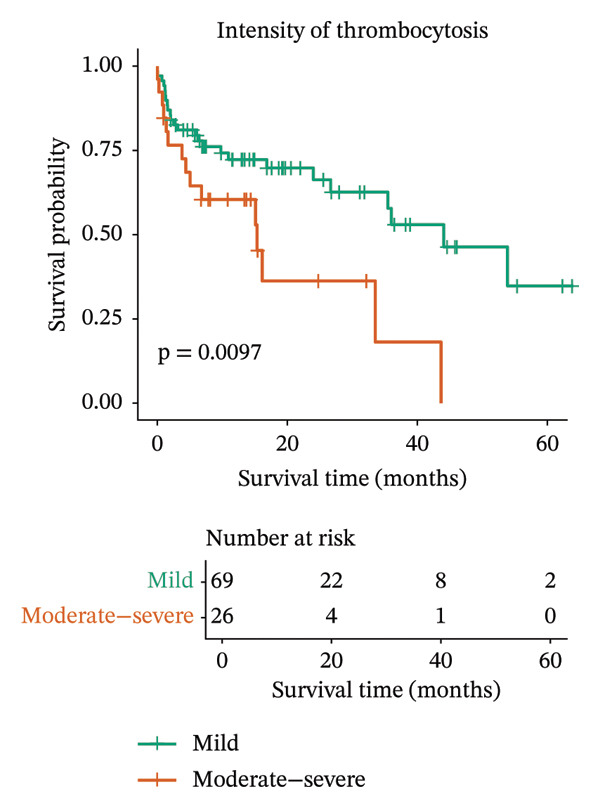
(b)

The same analysis was also performed considering only the cases diagnosed by histopathology because of better accuracy. This analysis revealed similar results (Figure 1(b)), indicating that the intensity of thrombocytosis could be a prognostic biomarker.

Multivariate Cox proportional hazards analysis was conducted to evaluate the prognostic impact of thrombocytosis intensity together with histogenesis and metastatic status. The model was applied both to all malignant cases (Table 1) and to the subset confirmed by histopathology (Table 2). In both analyses, moderate‐to‐severe thrombocytosis and the presence of metastasis were associated with a significantly worse prognosis.

**TABLE 1:** Multivariate Cox regression analysis of prognostic factors associated with survival in dogs with malignant neoplasms (*n* = 117; 57 events).

Factor	Factor level	HR	CI95% (lower–upper)	*p* value
Intensity of thrombocytosis	Mild (reference)	1.0	—	—
	Moderate to severe	2.17	1.212–3.886	0.009^∗∗^

Histogenesis	Epithelial (reference)	1.0	—	—
	Mesenchymal	1.91	1.017–3.602	0.044^∗^
	Round cell	2.637	1.268–5.485	0.009^∗∗^

Metastasis	Not detected (reference)	1.0	—	—
	Detected	2.62	1.507–4.574	< 0.001^∗∗∗^

Abbreviations: CI = confidence interval; HR = hazard ratio.

^∗^ *p* ≤ 0.05.

^∗∗^ *p* ≤ 0.01.

^∗∗∗^ *p* ≤ 0.001.

**TABLE 2:** Multivariate Cox regression analysis of prognostic factors associated with survival in dogs with malignant neoplasms diagnosed by histopathology only (*n* = 79; 35 events).

Factor	Factor level	HR	CI95% (lower–upper)	*p* value
Intensity of thrombocytosis	Mild (reference)	1.0	—	—
	Moderate to severe	2.52	1.10–5.74	0.0278^∗^

Histogenesis	Epithelial (reference)	1.0	—	—
	Mesenchymal	1.25	0.54–2.9	0.6
	Round cell	2.31	0.97–5.52	0.058

Metastasis	Not detected (reference)	1.0	—	—
	Detected	3.9	1.92–8.19	< 0.001^∗∗∗^

Abbreviations: CI = confidence interval; HR = hazard ratio.

^∗^ *p* ≤ 0.05.

^∗∗^ *p* ≤ 0.01.

^∗∗∗^ *p* ≤ 0.001.

For the analysis related to specific diagnoses, only the most prevalent neoplasms in our study were evaluated, representing 56% of all analyzed neoplasms: mammary carcinoma (*n* = 45), SCC (*n* = 14), mast cell tumor (*n* = 16), sarcoma (*n* = 11), and hemangiosarcoma (*n* = 10).

With respect to SCC, sarcoma, and hemangiosarcoma, the intensity of thrombocytosis did not significantly impact the survival time of the affected dogs in this study (Table S1). Moreover, the proportional hazards assumption was violated by thrombocytosis intensity in SCC and by metastatic status in sarcoma; therefore, these Cox regression findings should be interpreted with caution.

**FIGURE 2:**
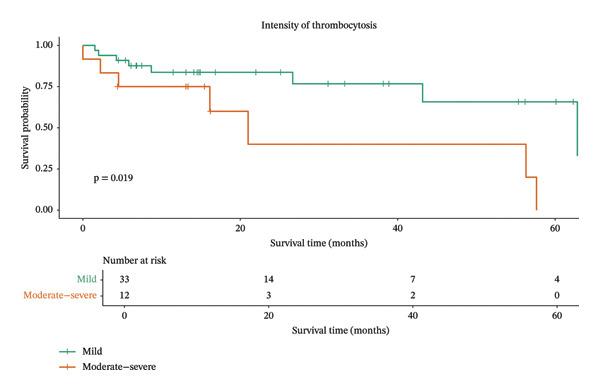
Kaplan‒Meier survival curves for dogs with mammary carcinoma stratified by the intensity of thrombocytosis. Patients with mild thrombocytosis (green line) exhibited a significantly longer survival time compared to those with moderate‐to‐severe thrombocytosis (orange line) (*p* = 0.019).

In contrast, animals diagnosed with mammary carcinoma and mild thrombocytosis had a more favorable prognosis according to the results of the Kaplan–Meier analysis, with a median survival of 62 months (95% CI: 43–not estimable). Conversely, those with moderate‐to‐severe thrombocytosis had a median survival of only 21 months (95% CI: 16–not estimable) (Figure 2). In the multivariate Cox proportional hazards model, which also accounted for metastatic status, the intensity of thrombocytosis emerged as a potential independent prognostic factor. Specifically, animals with moderate‐to‐severe thrombocytosis exhibited a more than threefold increased risk of death (HR 3.4, 95% CI: 1.16–9.83; *p* = 0.02) (Table 3).

**TABLE 3:** Multivariate Cox regression analysis of prognostic factors associated with survival in dogs with mammary carcinoma (*n* = 45; 15 events).

Factor	Factor level	HR	CI95% (lower–upper)	*p* value
Intensity of thrombocytosis	Mild (reference)	1.0	—	—
	Moderate to severe	3.40	1.16–9.83	0.02^∗^

Metastasis	Not detected (reference)	1.0	—	—
	Detected	4.04	1.25–13.10	0.01^∗∗^

Abbreviations: CI = confidence interval; HR = hazard ratio.

^∗^ *p* ≤ 0.05.

^∗∗^ *p* ≤ 0.01.

^∗∗∗^ *p* ≤ 0.001.

**FIGURE 3:**
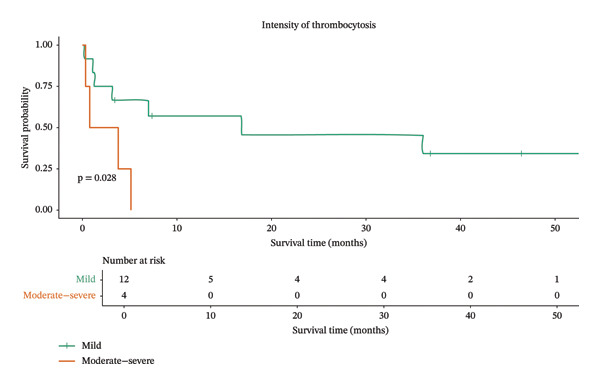
Kaplan‒Meier survival curves for dogs with mast cell tumors stratified by the intensity of thrombocytosis. Patients with mild thrombocytosis (green line) exhibited a significantly longer survival time compared to those with moderate‐to‐severe thrombocytosis (orange line) (*p* = 0.028).

In addition, animals with mast cell tumors and moderate‐to‐severe thrombocytosis had markedly shorter survival times (median 2.27 months; 95% CI: 0.3–not estimable), whereas those with mild thrombocytosis had longer survival times (median 16.8 months; 95% CI: 3.1–not estimable), suggesting that thrombocytosis intensity may play an important role in survival outcomes for these animals (Figure 3). However, in the multivariate Cox proportional hazards model, a trend toward significance was observed (HR 7.39; *p* = 0.052) (Table 4), indicating a possible prognostic impact that warrants further investigation.

**TABLE 4:** Multivariate Cox regression analysis of prognostic factors associated with survival in dogs with mast cell tumors (*n* = 13; 10 events).

Factor	Factor level	HR	CI95% (lower–upper)	*p* value
Intensity of thrombocytosis	Mild (reference)	1.0	—	—
	Moderate to severe	7.39	0.98–56.06	0.05

Metastasis	Not detected (reference)	1.0	—	—
	Detected	6.66	1.09–40.35	0.03^∗^

Abbreviations: CI = confidence interval; HR = hazard ratio.

^∗^ *p* ≤ 0.05.

^∗∗^ *p* ≤ 0.01.

^∗∗∗^ *p* ≤ 0.001.

## 4. Discussion

Our study represents one of the first investigations into the impact of thrombocytosis intensity on survival in dogs with malignant neoplasia. As an initial exploratory analysis, it opens avenues for future prospective studies with more robust methodologies and larger sample sizes; however, the observed results must be interpreted cautiously given the study’s limitations.

This study demonstrated a negative impact on the survival of dogs with malignant neoplasms because of the intensity of thrombocytosis. These findings suggest that the intensity of thrombocytosis may be an important prognostic factor in dogs diagnosed with malignant neoplasms. However, without a normal control group, it remains unclear whether thrombocytosis itself is a prognostic factor or whether only increasing intensity among thrombocytotic patients is linked to outcome differences.

In the multivariate Cox analysis, both the intensity of thrombocytosis and the presence of metastasis emerged as independent prognostic factors, regardless of the diagnostic methodology employed. Notably, compared with those with mild thrombocytosis, animals with moderate‐to‐severe thrombocytosis exhibited a more than twofold increase in the risk of death. This association mirrors findings in human medicine, where thrombocytosis is a well‐recognized prognostic factor for cancer patients [30–32]. The overall survival model may have been affected by tumor type heterogeneity. Therefore, an individual neoplasm subtype survival model was evaluated for each most common neoplasm in our study.

Bornardi [31] demonstrated that elevated platelet counts in preoperative exams tend to be useful for identifying patients with unfavorable prognosis. Furthermore, increased platelet counts have been revealed as a predictor of cancer in patients with occult malignancies [33].

In humans, as demonstrated by Balamurugan et al. [34], malignant neoplasms can benefit from angiogenic and tissue/cellular growth factors derived from platelets, such as PDGF, a component of platelet alpha granules that are secreted and act as potent proangiogenic factors [35].

PDGF is expressed in various cell types and malignant tumor tissues and has been shown to participate in proliferation, angiogenesis, migration, and invasion of many tumors, particularly malignant ones [34].

In human medicine, novel therapeutic methods targeting the PDGF pathway have emerged for cancer treatment since high PDGF expression in neoplasms can lead to therapeutic resistance, whereas its inhibition suppresses cancer proliferation, metastasis, invasion, and angiogenesis [36]. While most drugs have proven effective and well tolerated, others have shown lower efficacy and displayed potential toxicity in clinical trials [37].

Furthermore, as we observed, metastatic status is a well‐established prognostic factor. However, we must acknowledge a limitation in our study: only 10 cases were confirmed by histopathology, while the remainder relied on diagnostic imaging. Although positive imaging findings are reliable, the absence of detection should be interpreted with caution, as the sensitivity of these modalities ranges from 65% to 97% [38]. Mammary carcinoma was the most frequent diagnosis associated with thrombocytosis in our study. Similar findings were reported by Neel et al. [25] and Ribas et al. [39], who reported that thrombocytosis is more prevalent than thrombocytopenia in female dogs with mammary carcinoma and adenocarcinoma.

Furthermore, more than half of the mammary carcinoma diagnoses in our study were obtained through cytopathological examination, a technique known to have lower accuracy than the histopathological type of neoplasia [40]. However, a reassessment using only histopathological confirmed mammary carcinomas (*n* = 22) yielded similar results in the Kaplan‒Meier analysis (Table S1), although the statistical power was limited because of the small number of events (*n* = 4/22).

Kaplan‒Meier analysis revealed that patients with mammary carcinoma and moderate‐to‐marked thrombocytosis had shorter survival times than those with mild thrombocytosis did. This may be attributed to the ability of tumor cells to directly activate platelets, promoting aggregation and the release of growth factors. These processes facilitate tumor growth and angiogenesis, contributing to neoplastic progression and, consequently, reduced survival [41].

Furthermore, another mechanism proposed in human cancer involves the release of IL‐6 by tumor cells, which stimulates hepatic TPO production and consequently contributes to thrombocytosis [33].

However, Cheney et al. [42], evaluating platelet counts and serum TPO and IL‐6 concentrations in dogs with various carcinomas, did not identify increased IL‐6 levels compared with those in healthy dogs or differences between carcinoma subgroups.

Conversely, TPO concentrations were significantly higher in dogs with carcinoma, independent of platelet count, suggesting that TPO is one of several factors regulating platelet production in canine cancer. It is important to note, however, that this study did not evaluate mammary carcinoma and included animals with comorbidities, such as renal disease, endocrine disorders, and hepatopathies, which can influence platelet counts both positively and negatively [42]. These parameters need to be evaluated in specific neoplasms to determine whether such mechanisms are present in canine tumors.

Survival times for female dogs with mammary carcinoma vary widely in the literature, ranging from a few months to more than 2 years [43–46]. Prognosis is strongly associated with clinical stage, histological subtype, tumor grade, and surgical margin status; however, these variables were not included in our Cox analysis. Consequently, further studies are warranted to evaluate the interactions between these established prognostic factors and platelet count to determine whether thrombocytosis intensity is an independent prognostic factor or reflects more aggressive tumor biology, such as high‐grade tumors or clinical stage.

Mast cell tumors were the most prevalent round cell neoplasms in our study (69.5%). Kaplan‒Meier analysis demonstrated that the intensity of thrombocytosis significantly influenced survival (*p* = 0.028); notably, dogs with moderate‐to‐marked thrombocytosis exhibited a reduced survival time of approximately 2.27 months. These findings are further supported by Cox regression, which revealed a trend (*p* = 0.05), indicating that these patients had a 7.39‐fold increased risk of death (HR = 7.39; 95% CI: 0.98–56.06), suggesting that thrombocytosis may serve as a valuable additional marker for poor prognosis, although the wide confidence intervals associated with the small sample size (*n* = 16) in the multivariate Cox regression represent a limitation that should be interpreted with caution.

Currently, the literature presents conflicting results regarding the prevalence of thrombocytosis in dogs with mast cell tumors. For example, in the study by Amaral et al. [47], five out of six patients with mast cell tumors presented platelet counts within the reference range, with only one case presenting discrete thrombocytosis.

Conversely, in studies by Neel et al. [25] and Hammer et al. [24], thrombocytosis was observed primarily in round cell neoplasms, including mast cell tumors. However, the dogs in these studies were under corticosteroid treatment, a medication known to increase platelet counts by reducing macrophage phagocytosis [29], which is frequently used in the clinical treatment of cutaneous mast cell tumors [48].

On the other hand, none of these studies assessed the impact of thrombocytosis on the prognosis of these patients. This highlights the need for further research to understand the prognostic relationship between thrombocytosis and canine mast cell tumors. Such research is particularly important since mast cell tumors are among the most common types of cancer worldwide [49, 50].

High‐grade cutaneous mast cell tumors are recognized as highly aggressive, with a median survival of less than 4 months, whereas low‐grade tumors tend to present survival exceeding 2 years [51]. Given its low cost and accessibility, platelet counting could improve the clinical stratification of high‐risk patients, as evaluating thrombocytosis intensity alongside tumor grading could offer significant prognostic value for cutaneous mast cell tumors. However, in the present study, tumor grades were not evaluated, as the cohort included patients diagnosed by both histopathology and cytopathology. Consequently, future research is needed to clarify whether moderate‐to‐marked thrombocytosis reflects a high histological grade or constitutes an independent prognostic factor.

Sarcomas and hemangiosarcomas were not significantly associated with a specific diagnostic group, which may have been due to our methodology, in which soft tissue tumors and poorly differentiated sarcomas from different locations (e.g., cutaneous, ocular, and bone tissues) were all grouped under the same sarcoma diagnosis.

Although tumors should ideally be classified on the basis of histopathological and/or immunohistochemical findings and categorized according to their anatomical location, the limited number of cases in our study prevented this assessment from being conducted separately. Therefore, the results obtained may present some uncertainties, highlighting the need for further studies to achieve a more precise prognostic evaluation.

Abou et al. [52] reported that dogs with hemangiosarcoma exhibit high expression of platelet‐derived growth factor receptor (PDGFR) types, particularly PDGFR‐β, which is commonly associated with tumor progression.

The activation of PDGFRs can trigger molecular events such as cellular growth stimulation, actin rearrangement, and the inhibition of intercellular communication, all of which are essential processes for modulating cellular responses and, consequently, contribute to tumor progression [53].

The life expectancy of dogs diagnosed with hemangiosarcoma is influenced by the location of the tumor, with significant variations depending on the affected organ [54]. In our study, hemangiosarcoma was predominantly observed in the cutaneous region (9/10 cases), with only one case identified in the spleen.

The biological behavior of nonvisceral hemangiosarcoma is influenced by tumor location (cutaneous, subcutaneous, and muscular) and the degree of tissue invasion. Clinical stage is a crucial prognostic factor for determining disease progression [54].

In a retrospective study by Szivek et al. [55], dogs diagnosed with cutaneous hemangiosarcoma who underwent surgery exclusively had a median survival of 25–32 months. In contrast, dogs with splenic hemangiosarcoma treated with splenectomy have a median survival time of 1 year and 6 months [56].

Therefore, further studies are needed to better understand the influence of platelet count in animals with cutaneous hemangiosarcoma because platelet consumption occurs in these tumors.

No significant association was detected between the intensity of thrombocytosis and survival in dogs with SCC, likely because of the limited sample size and the heterogeneity of tumor sites in the present study. Conversely, in human medicine, studies on head, neck, and oral SCCs have indicated that elevated pretreatment platelet counts and increased platelet‐to‐lymphocyte ratios are associated with poor survival, although these markers do not consistently remain independent prognostic factors across all cohorts [57, 58]. These findings suggest that platelets and hematology‐derived inflammatory indices may play a relevant role in SCC behavior, underscoring the need for further studies with larger sample sizes and stratification by tumor site and stage to clarify their prognostic impact in canines.

Although medical records were rigorously screened to exclude patients with clinical or laboratory evidence of endocrine disorders, metabolic diseases, or other conditions potentially causing relevant hemostatic alterations, thereby minimizing reactive thrombocytosis, the inclusion of quantitative inflammatory markers (e.g., differential leukocyte counts, neutrophil‐to‐lymphocyte ratios, and acute‐phase proteins) would be ideal to fully rule out this bias. Prospective studies incorporating serial assessments of these biomarkers are warranted to adequately isolate their confounding effects and validate the observed associations.

## 5. Conclusion

The intensity of thrombocytosis represents a promising prognostic indicator for dogs with malignant neoplasms, particularly in cases of mammary carcinoma and cutaneous mast cell tumors. While these results should be viewed alongside the lack of a normoplatelet control group and the limited adjustment for all established prognostic markers, they highlight a significant clinical trend. Future research will validate thrombocytosis as a prognostic biomarker and elucidate its role in tumor biology and clinical outcomes in veterinary oncology.

## Author Contributions

Conceptualization, L.G.S. and R.K.T.; methodology, L.G.S. and R.K.T.; formal analysis, F.S.T., L.G.S., and R.K.T.; investigation, L.G.S.; resources, L.G.S. and R.K.T.; writing–original draft preparation, L.G.S.; writing–review and editing, LG.S., F.S.T., S.E.V., and R.K.T.; supervision, R.K.T.; and project administration, R.K.T.

## Funding

No funding was received for this manuscript.

## Disclosure

All authors have read and agreed to the published version of the manuscript.

## Conflicts of Interest

The authors declare no conflicts of interest.

## Supporting Information

Additional supporting information can be found online in the Supporting Information section.

## Supporting information


**Supporting Information** Supporting data: Table S1. Raw data table of animals, including diagnosis, histogenesis, biological behavior, platelet count, metastatic status, and survival time. Table S2. Survival analysis results. Kaplan–Meier and Cox regression outputs for all major neoplasms.

## Data Availability

The data that support the findings of this study are available in the supporting material of this article.
