# Supplementary material for: Investigation of the Intensity of Thrombocytosis as a Potential Prognostic Indicator in Canine Malignant Neoplasms
Source: Vet Med Int. 2026 May 21;2026:1271185. doi: 10.1155/vmi/1271185 (PMC13195024; doi:10.1155/vmi/1271185)
Supplement: Supplementary file 1 — Supporting Information Supporting data: Table S1. Raw data table of animals, including diagnosis, histogenesis, biological behavior, platelet count, metastatic status, and survival time. Table S2. Survival analysis results. Kaplan–Meier and Cox regression outputs for all major neoplasms. [file VMI-2026-1271185-s001.zip › Supplementary-Document.docx]

Supplementary Document

# Overall Malignancy

### Median Survival Time

**Median Survival Time according to Thrombocytosis Intensity**

| **Characteristic** | **Median Survival (months)** |
| --- | --- |
| Intensity |  |
| Mild | 44 (35, —) |
| Moderate-severe | 15 (4.5, 56) |

### Kaplan-Meier Curve


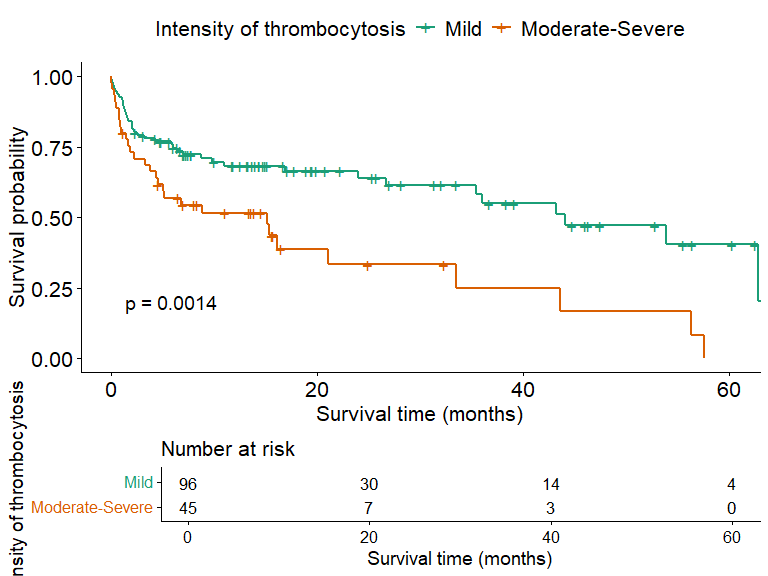


### Multivariate Cox Model

**Multivariate Cox Proportional Hazards Model**

| **Characteristic** | **HR** | **95% CI** | **p-value** |
| --- | --- | --- | --- |
| Intensity |  |  |  |
| Mild | — | — |  |
| Moderate-severe | 2.17 | 1.21, 3.89 | **0.009** |
| Histogenesis |  |  |  |
| Epithelial | — | — |  |
| Mesenchymal | 1.91 | 1.02, 3.60 | **0.044** |
| Round cell | 2.64 | 1.27, 5.49 | **0.009** |
| meta_status |  |  |  |
| No evidence | — | — |  |
| Detected | 2.63 | 1.51, 4.57 | **<0.001** |
| Abbreviations: CI = Confidence Interval, HR = Hazard Ratio | | | |

### Schoenfeld Test

Schoenfeld Residuals Test

|  | chisq | df | p |
| --- | --- | --- | --- |
| Intensity | 1.710 | 1 | 0.191 |
| Histogenesis | 0.229 | 2 | 0.892 |
| meta_status | 0.014 | 1 | 0.904 |
| GLOBAL | 1.795 | 4 | 0.773 |

# Overall malignancy - Histopathology Exams Only

### Median Survival Time

**Median Survival Time (Histopathology Only)**

| **Characteristic** | **Median Survival (months)** |
| --- | --- |
| Intensity |  |
| Mild | 44 (27, —) |
| Moderate-severe | 15 (5.0, —) |

### Kaplan-Meier Curve


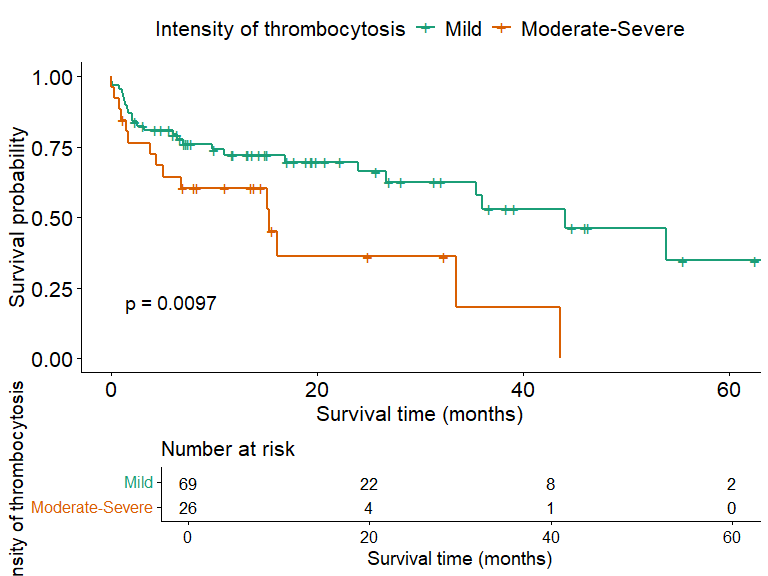


### Multivariate Cox Model

**Multivariate Cox Model (Histopathology Only)**

| **Characteristic** | **HR** | **95% CI** | **p-value** |
| --- | --- | --- | --- |
| Intensity |  |  |  |
| Mild | — | — |  |
| Moderate-severe | 2.52 | 1.11, 5.75 | **0.028** |
| Histogenesis |  |  |  |
| Epithelial | — | — |  |
| Mesenchymal | 1.25 | 0.54, 2.89 | 0.6 |
| Round cell | 2.31 | 0.97, 5.52 | 0.059 |
| meta_status |  |  |  |
| No evidence | — | — |  |
| Detected | 3.96 | 1.92, 8.19 | **<0.001** |
| Abbreviations: CI = Confidence Interval, HR = Hazard Ratio | | | |

### Schoenfeld Test

|  | chisq | df | p |
| --- | --- | --- | --- |
| Intensity | 1.228 | 1 | 0.268 |
| Histogenesis | 1.279 | 2 | 0.528 |
| meta_status | 0.581 | 1 | 0.446 |
| GLOBAL | 2.958 | 4 | 0.565 |

# Analysis by Prevalent Diagnosis

## 1. Mammary Carcinoma

### Median Survival Time

**Mammary Carcinoma Median Survival**

| **Characteristic** | **Median Survival (months)** |
| --- | --- |
| Intensity |  |
| Mild | 63 (43, —) |
| Moderate-severe | 21 (16, —) |

### Kaplan-Meier Curve


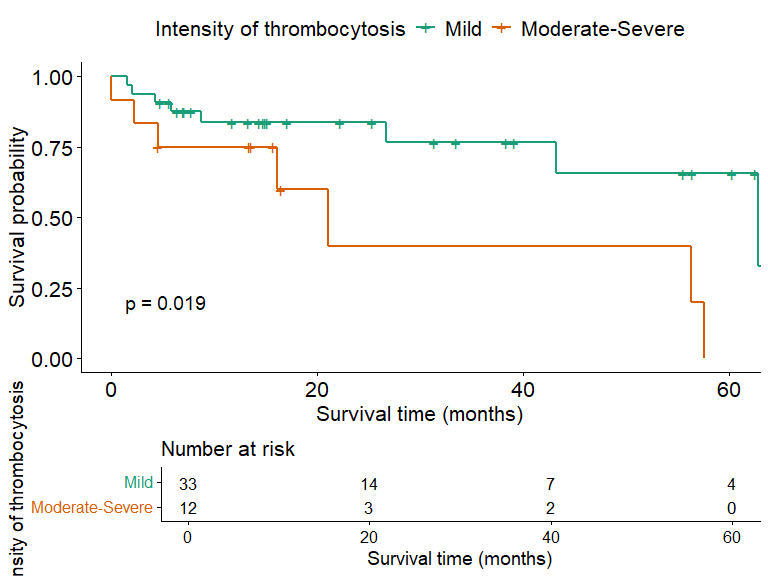


### Multivariate Cox Model

**Mammary Carcinoma Multivariate Cox Model**

| **Characteristic** | **HR** | **95% CI** | **p-value** |
| --- | --- | --- | --- |
| Intensity |  |  |  |
| Mild | — | — |  |
| Moderate-severe | 3.38 | 1.16, 9.84 | **0.025** |
| meta_status |  |  |  |
| No evidence | — | — |  |
| Detected | 4.04 | 1.25, 13.1 | **0.020** |
| Abbreviations: CI = Confidence Interval, HR = Hazard Ratio | | | |

### Schoenfeld Test

|  | chisq | df | p |
| --- | --- | --- | --- |
| Intensity | 1.378 | 1 | 0.240 |
| meta_status | 1.837 | 1 | 0.175 |
| GLOBAL | 3.384 | 2 | 0.184 |

## 2. Squamous Cell Carcinoma

### Median Survival Time

**Squamous Cell Carcinoma Median Survival**

| **Characteristic** | **Median Survival (months)** |
| --- | --- |
| Intensity |  |
| Mild | 35 (2.0, —) |
| Moderate-severe | 8.8 (6.8, —) |

### Kaplan-Meier Curve


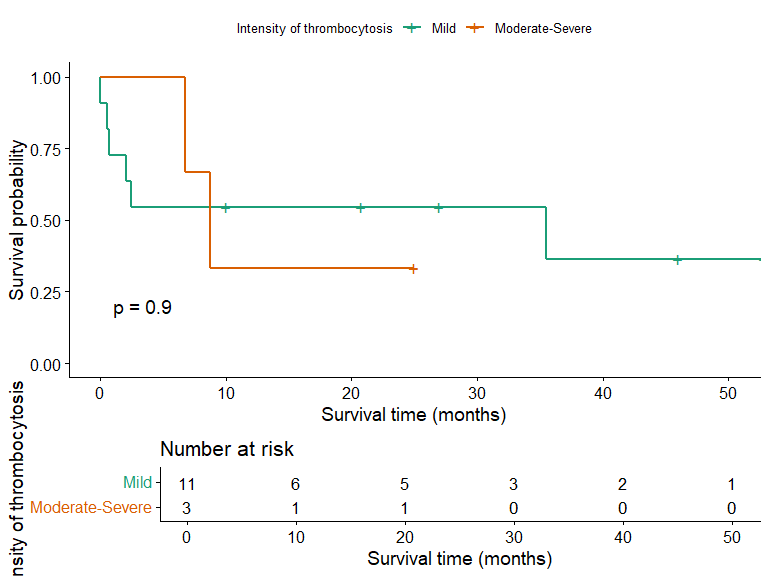


### Multivariate Cox Model

**Squamous Cell Carcinoma Multivariate Cox Model**

| **Characteristic** | **HR** | **95% CI** | **p-value** |
| --- | --- | --- | --- |
| Intensity |  |  |  |
| Mild | — | — |  |
| Moderate-severe | 0.93 | 0.17, 5.18 | >0.9 |
| meta_status |  |  |  |
| No evidence | — | — |  |
| Detected | 2.15 | 0.47, 9.74 | 0.3 |
| Abbreviations: CI = Confidence Interval, HR = Hazard Ratio | | | |

### Schoenfeld Test

|  | chisq | df | p |
| --- | --- | --- | --- |
| Intensity | 4.052 | 1 | 0.044 |
| meta_status | 0.618 | 1 | 0.432 |
| GLOBAL | 4.776 | 2 | 0.092 |

## 3. Mast Cell Tumor

### Median Survival Time

**Mast Cell Tumor Median Survival**

| **Characteristic** | **Median Survival (months)** |
| --- | --- |
| Intensity |  |
| Mild | 17 (3.1, —) |
| Moderate-severe | 2.3 (0.33, —) |

### Kaplan-Meier Curve


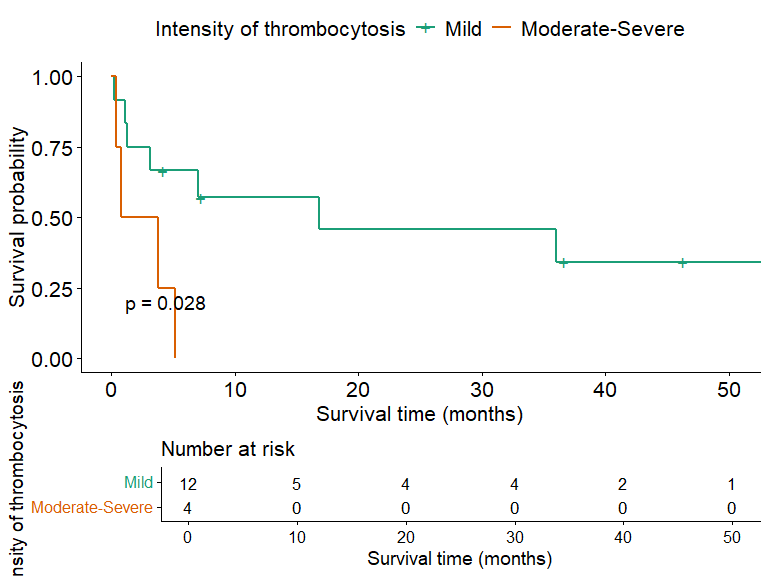


### Multivariate Cox Model

**Mast Cell Tumor Multivariate Cox Model**

| **Characteristic** | **HR** | **95% CI** | **p-value** |
| --- | --- | --- | --- |
| Intensity |  |  |  |
| Mild | — | — |  |
| Moderate-severe | 7.40 | 0.98, 56.1 | 0.053 |
| meta_status |  |  |  |
| No evidence | — | — |  |
| Detected | 6.66 | 1.10, 40.3 | **0.039** |
| Abbreviations: CI = Confidence Interval, HR = Hazard Ratio | | | |

### Schoenfeld Test

|  | chisq | df | p |
| --- | --- | --- | --- |
| Intensity | 0.263 | 1 | 0.608 |
| meta_status | 0.001 | 1 | 0.971 |
| GLOBAL | 0.327 | 2 | 0.849 |

## 4. Hemangiosarcoma

### Median Survival Time

**Hemangiosarcoma Median Survival**

| **Characteristic** | **Median Survival (months)** |
| --- | --- |
| Intensity |  |
| Mild | — (—, —) |
| Moderate-severe | 9.8 (1.4, —) |

### Kaplan-Meier Curve


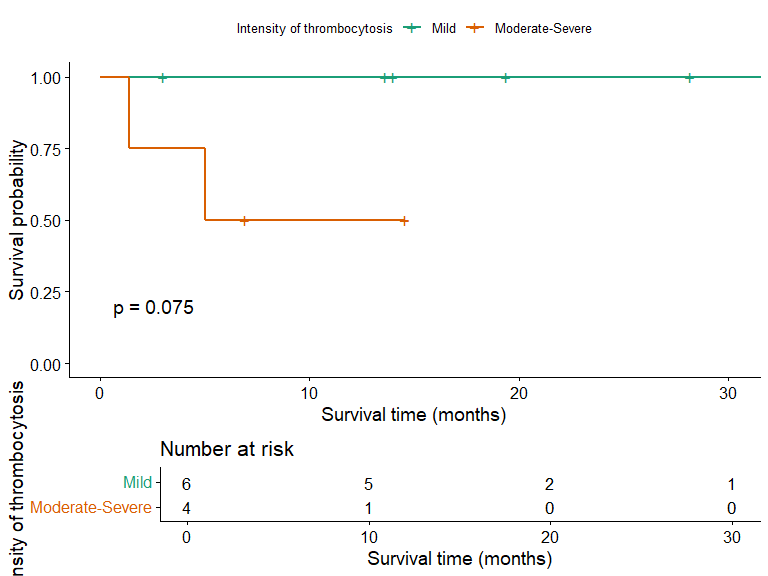


### Multivariate Cox Model

**Hemangiosarcoma Multivariate Cox Model**

| **Characteristic** | **HR** | **95% CI** | **p-value** |
| --- | --- | --- | --- |
| Intensity |  |  |  |
| Mild | — | — |  |
| Moderate-severe | 3,322,816,878 | 0.00, Inf | >0.9 |
| meta_status |  |  |  |
| No evidence | — | — |  |
| Detected | 0.71 | 0.04, 11.8 | 0.8 |
| Abbreviations: CI = Confidence Interval, HR = Hazard Ratio | | | |

### Schoenfeld Test

|  | chisq | df | p |
| --- | --- | --- | --- |
| Intensity | 0.000 | 1 | 1.000 |
| meta_status | 1.414 | 1 | 0.234 |
| GLOBAL | 1.414 | 2 | 0.493 |

## 5. Sarcoma

### Median Survival Time

**Sarcoma Median Survival**

| **Characteristic** | **Median Survival (months)** |
| --- | --- |
| Intensity |  |
| Mild | — (1.4, —) |
| Moderate-severe | 1.4 (0.77, —) |

### Kaplan-Meier Curve


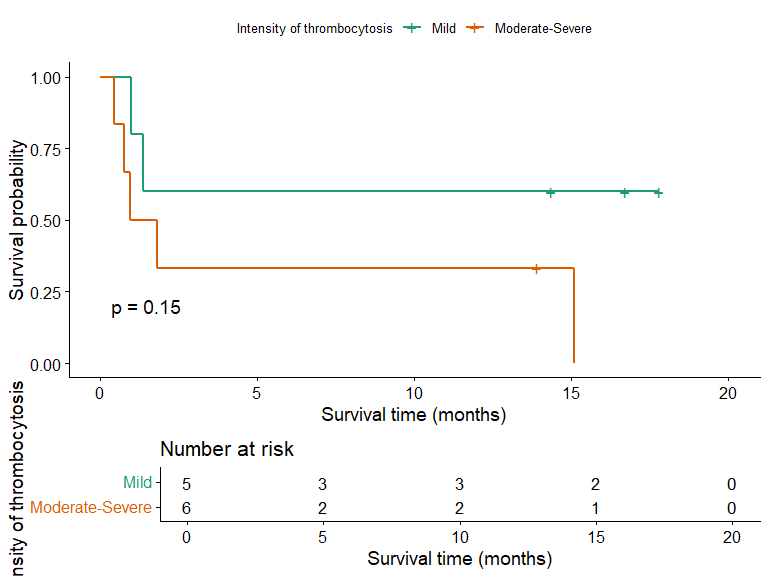


### Multivariate Cox Model

**Sarcoma Multivariate Cox Model**

| **Characteristic** | **HR** | **95% CI** | **p-value** |
| --- | --- | --- | --- |
| Intensity |  |  |  |
| Mild | — | — |  |
| Moderate-severe | 2.74 | 0.50, 14.9 | 0.2 |
| meta_status |  |  |  |
| No evidence | — | — |  |
| Detected | 2.20 | 0.43, 11.4 | 0.3 |
| Abbreviations: CI = Confidence Interval, HR = Hazard Ratio | | | |

### Schoenfeld Test

|  | chisq | df | p |
| --- | --- | --- | --- |
| Intensity | 0.026 | 1 | 0.872 |
| meta_status | 3.378 | 1 | 0.066 |
| GLOBAL | 3.607 | 2 | 0.165 |
